# Supplementary material for: Asynchronous magnetic resonance elastography: Shear wave speed reconstruction using noise correlation of incoherent waves
Source: Magn Reson Med. 2022 Oct 27;89(3):990–1001. doi: 10.1002/mrm.29502 (PMC9792433; doi:10.1002/mrm.29502)
Supplement: Supplementary file 1 — DATA S1 MATLAB code used to generate the numerical simulations of Figure 1 [file MRM-89-990-s002.zip › k-Wave/helpfiles/example_tvsp_steering_linear_array.html]

Steering A Linear Array Example (k-Wave)


# Steering A Linear Array Example

This example demonstrates how to use k-Wave to steer a tone burst from a linear array transducer in 2D. It builds on the Simulating Transducer Field Patterns Example.

- Open the file in the MATLAB Editor
- Run the file in MATLAB

## Contents

- Defining the linear array and input signal
- Running the simulation

## Defining the linear array and input signal

The source mask is defined by creating a binary mask with the desired source points set to 1.

```
% define source mask for a linear transducer with an odd number of elements   
num_elements = 21;      % [grid points]
x_offset = 25;          % [grid points]
source.p_mask = zeros(Nx, Ny);
start_index = Ny/2 - round(num_elements/2) + 1;
source.p_mask(x_offset, start_index:start_index + num_elements - 1) = 1;
```

The input signal to each element is then created using
the function `toneBurst` with a geometrically steered temporal offset that varies across the source.

```
% define the properties of the tone burst used to drive the transducer
sampling_freq = 1/kgrid.dt;     % [Hz]
steering_angle = 30;            % [deg]
element_spacing = dx;           % [m]
tone_burst_freq = 1e6;          % [Hz]
tone_burst_cycles = 8;

% create an element index relative to the centre element of the transducer
element_index = -(num_elements - 1)/2:(num_elements - 1)/2;

% use geometric beam forming to calculate the tone burst offsets for each
% transducer element based on the element index
tone_burst_offset = 40 + element_spacing * element_index * ...
    sin(steering_angle * pi/180) / (medium.sound_speed * kgrid.dt);

% create the tone burst signals
source.p = toneBurst(sampling_freq, tone_burst_freq, tone_burst_cycles, ...
    'SignalOffset', tone_burst_offset);
```

A plot of the input signals for each transducer element is shown below.


## Running the simulation

Visualisations of the pressure field at two different times for steering angles of +30 and -20 degrees are shown below.
